# A collaborative multi-attention network for real-time small object detection in UAV imagery

**DOI:** 10.1038/s41598-026-36440-2

**Published:** 2026-01-20

**Authors:** Jianxiu Yang, Xiangmei Yue, Liang Wu

**Affiliations:** School of Physics and Electronics, Shanxi Datong University, Datong, 037009 China

**Keywords:** Attention mechanism, Dual-dimensional channels, Foreground features, Small object detection, Engineering, Mathematics and computing

## Abstract

To address the challenges of detecting small objects in unmanned aerial vehicle (UAV) imagery, such as weak feature representation, complex background interference, and high real-time requirements, this paper proposes a Collaborative Multi-Attention Network (CMA-Net) for real-time small object detection. The network incorporates an efficient bi-directional feature pyramid structure (E-BiFPN) to achieve multi-scale weighted feature fusion while minimizing parameter count and computational cost. A Dual-Dimensional Channel Attention (DDCA) module is further introduced, which adaptively recalibrates channel significance along the width and height dimensions, capturing long-range dependencies and improving spatial sensitivity. Additionally, a Multi-Scale Foreground Attention (MSFA) module is designed to explore inter-object correlations across different feature layers, enhancing foreground representation, suppressing background interference, and improving feature discriminability for small objects. By integrating E-BiFPN, DDCA, and MSFA, CMA-Net achieves collaborative feature enhancement and significantly boosts overall discriminative power. Experimental results demonstrate that the proposed method achieves accuracies of 67.2% and 62.0% on the public UAVDT and Stanford Drone datasets, respectively, while operating at 64 frames per second, meeting real-time inference requirements.

## Introduction

Small object detection is a fundamental and challenging task in computer vision, with significant applications in areas such as UAV-based intelligent surveillance^1,2^ and remote sensing^3,4^. This study focuses specifically on the detection of multi-class vehicles from UAV imagery. Owing to the extensive field of view and highly variable viewpoints characteristic of UAV platforms, the captured scenes are often subject to complex environmental conditions and variable lighting. These factors result in targets that are small in size and low in resolution, thereby complicating feature extraction and increasing detection difficulty. In practical UAV applications, where onboard communication constraints must be considered^5,6^, achieving both high accuracy and real-time performance is critical. This makes the design of detection methods that effectively balance accuracy with computational efficiency a matter of considerable practical importance.

With the advancement of deep learning, two-stage detectors such as Faster R-CNN^7^ and Cascade R-CNN^8^, which achieve high detection accuracy at the cost of computational efficiency, thus limiting their real-time applicability. In contrast, single-stage detectors like YOLO series^9–11^ offer faster inference speeds but often deliver inferior performance for small objects in UAV imagery. A primary reason for this shortcoming is that general object detection algorithms often do not pay enough attention to small objects, resulting in the progressive loss of small object information during feature transmission, weakened feature expression, and ultimately, inaccurate detection. To enhance the feature representation of small objects, several effective strategies have been developed, primarily based on feature fusion and attention mechanisms. Feature fusion typically relies on classical feature pyramid networks (FPN)^12^, which facilitate multi-scale feature integration. However, the conventional top-down FPN architecture predominantly enriches semantic context beneficial for classification, while providing limited spatial information necessary for precise localization. To mitigate this limitation, bidirectional feature pyramid networks such as Bi-FPN^13^ and PANet^14^ have been introduced, incorporating both top-down and bottom-up pathways to effectively aggregate complementary multi-scale features.

Building upon these advances, we propose an Efficient Bi-directional Feature Pyramid Network (E-BiFPN) specifically designed to overcome the core limitations of UAV-based detection. To achieve real-time performance, the network incorporates a lightweight FBlock built with partial convolution, which substantially lowers both the parameter count and computational complexity. Furthermore, a novel Weighted Feature Fusion Mechanism (WFFM) is introduced to adaptively recalibrate multi-scale features, emphasizing shallow details and suppressing irrelevant background interference. This unified design enables the network to capture essential contextual information for recognition while preserving fine-grained spatial details for precise localization, ultimately achieving both high accuracy and practical inference speed for aerial small object detection.

The attention mechanism, which mimics human cognitive processes, enables deep models to dynamically prioritize salient image regions by weighting spatial or channel features. For small object detection in UAV imagery, this proves especially critical, as it guides the model to amplify subtle yet discriminative features of tiny targets. Existing approaches^15–17^ typically embed attention modules within individual feature scales to capture intra-scale correlations. However, such single-scale attention fails to model the inherent relationships among objects that appear at different resolutions. To overcome this limitation, we propose a Multi-Scale Foreground Attention (MSFA) module. MSFA explicitly builds cross-scale foreground correlations by attending to and fusing multi-scale features from the E-BiFPN. This allows small targets to be enhanced with high-level semantic context from deeper layers while suppressing background interference, thereby strengthening inter-scale coherence and significantly boosting discriminative power for small object detection.

Furthermore, prevalent channel attention mechanisms^18–20^ typically compress spatial information into a single global descriptor, which inevitably diminishes the precise spatial cues vital for locating small objects in cluttered UAV scenes. To overcome this, we introduce a Dual-Dimensional Channel Attention (DDCA) module. Instead of global pooling, DDCA decomposes channel attention into separate width and height branches, performing adaptive recalibration along each spatial dimension. This design captures long-range dependencies in both directions, preserving accurate locational information while emphasizing semantically meaningful channels. The resulting features maintain heightened spatial sensitivity, effectively enhancing the discriminative representation of subtle small objects against complex backgrounds. By integrating DDCA with the previously described MSFA through feature fusion, our model jointly captures cross-scale object relationships and cross-dimensional channel dependencies. This synergistic combination is unified with the E-BiFPN backbone into our final architecture, the Collaborative Multi-Attention Network (CMA-Net), which progressively refines features to achieve robust and real-time small object detection in UAV imagery.

The main contributions of this paper can be summarized as follows:

1. An E-BiFPN is introduced to achieve efficient multi-scale feature fusion. It incorporates a lightweight FBlock with partial convolution to optimize speed and a novel WFFM to adaptively enhance detail-rich shallow features, thereby balancing high accuracy with real-time inference speed for UAV small object detection.
2. A DDCA module is proposed to recalibrate channel-wise features by exploiting inter-channel dependencies along both spatial dimensions (width and height), thereby enhancing spatial sensitivity and the representation of small objects.
3. A MSFA module is introduced to capture inter-object correlations across different scales. It enhances foreground representation while suppressing background interference, thereby improving the discriminative capability for small objects.

Experiments on UAVDT^21^ and Stanford Drone^22^ datasets show that the proposed method achieves state-of-the-art accuracy while maintaining real-time performance.

## Related work

### Small object detection in UAV imagery

Driven by the rapid development of convolutional neural networks (CNNs), small object detection in UAV imagery has been primarily approached through two-stage and single-stage detection frameworks. Two-stage detectors, often built upon architectures like Faster R-CNN and Cascade R-CNN, enhance feature representation to improve detection accuracy. Huang et al.^23^ propose UFPMP-Det that leverages a two-stage strategy: a Faster R-CNN^7^ first generates coarse region proposals, followed by a RetinaNet^24^ that performs the final detection on the merged sub-regions. Huang et al.^25^ utilize an improved Cascade R-CNN^8^ that incorporates a superclass detection head, fuses regression confidences, and modifies the loss function to boost detection performance. Xie et al.^26^ propose a density-guided two-stage framework that uses a dense object region seeker to focus on high-density areas and introduces the normalized Wasserstein distance loss to enhance bounding box regression accuracy. Despite their high accuracy, these two-stage methods often suffer from high computational complexity and limited inference speed. To meet the demands of real-time applications, several single-stage UAV object detection methods^27–29^ have been developed based on YOLO series, achieving high detection accuracy. However, a common limitation of these early single-stage approaches is their insufficient capacity to model the discriminative features of small objects. To address this, recent researches have increasingly focused on enhancing small object feature representation through advanced feature fusion and attention mechanisms.

### Feature fusion mechanisms

Effective feature fusion is crucial for multi-scale object detection, particularly for small objects. FPN^12^ established a foundational architecture by introducing a top-down pathway to integrate high-level semantic features with low-level spatial details. This design was later enhanced by subsequent innovations such as PANet^14^ and BiFPN^13^, which incorporated additional bottom-up paths to further strengthen information flow across scales. In the context of UAV-based small object detection, Zhang et al.^19^ introduce a BiFPN to enhance multi-scale feature fusion, leading to significantly improved detection of small targets. Bie et al.^30^ propose a bidirectional feature pyramid network to enhance multi-scale feature representation. By strategically combining features from different network levels through bidirectional connections, their method effectively enriches the feature hierarchy, leading to improved representation of small objects. Nian et al.^31^ propose a feature fusion module for adjacent layers, specifically designed to address the challenges of detecting small and occluded objects. Kiobya et al.^32^ propose a module that enhances multi-scale representation by transferring fine-grained semantic information from higher feature pyramid layers to the lowest one, specifically for improving small object detection. Zhu et al.^33^ propose a Gather-and-Distribute mechanism that enhances detection performance by effectively aggregating and redistributing multi-scale features. Inspired by these advances, we propose an efficient bi-directional feature pyramid network to achieve multi-scale weighted feature fusion with reduced computational cost.

### Attention mechanisms

Attention mechanisms have become an essential component in computer vision, enabling models to amplify critical features while suppressing irrelevant ones, thereby enhancing feature discriminability. The Squeeze-and-Excitation (SE) network^34^ pioneered channel attention, adaptively recalibrating channel-wise feature responses. Subsequent work like CBAM^35^ extended this by sequentially applying both channel and spatial attention. For UAV small object detection, Li et al.^17^ employ attention progressively from local patches to global contexts via a unified descriptor, enabling comprehensive modeling of both long-range dependencies and fine-grained details. Hamzenejadi et al.^36^ incorporate a novel channel attention mechanism, building upon the SE design, into the backbone network to enhance feature representation for real-time vehicle detection in UAV imagery. Wei et al.^37^ introduce a scale-aware feature amalgamation component preceding the detection head. It employs a scale attention mechanism to dynamically weight features from adjacent layers, thereby enhancing information flow and robustness against complex backgrounds and scale variations. Wang et al.^38^ introduce a coordinate spatial attention mechanism to enhance the capture of spatial and positional cues essential for small targets, thereby improving detection accuracy. Further advances include hybrid attention approaches that integrate both channel and spatial mechanisms^39,40^, effectively enhancing vehicle information while suppressing background clutter. For small object detection, it is crucial to preserve spatial details while modeling channel context, a capability limited in single-attention approaches. Our proposed collaborative multi-attention network addresses this gap by simultaneously capturing cross-scale object relationships and cross-dimensional channel correlations, thus synergistically enhancing feature representation.

## Proposed method

**Fig. 1:**
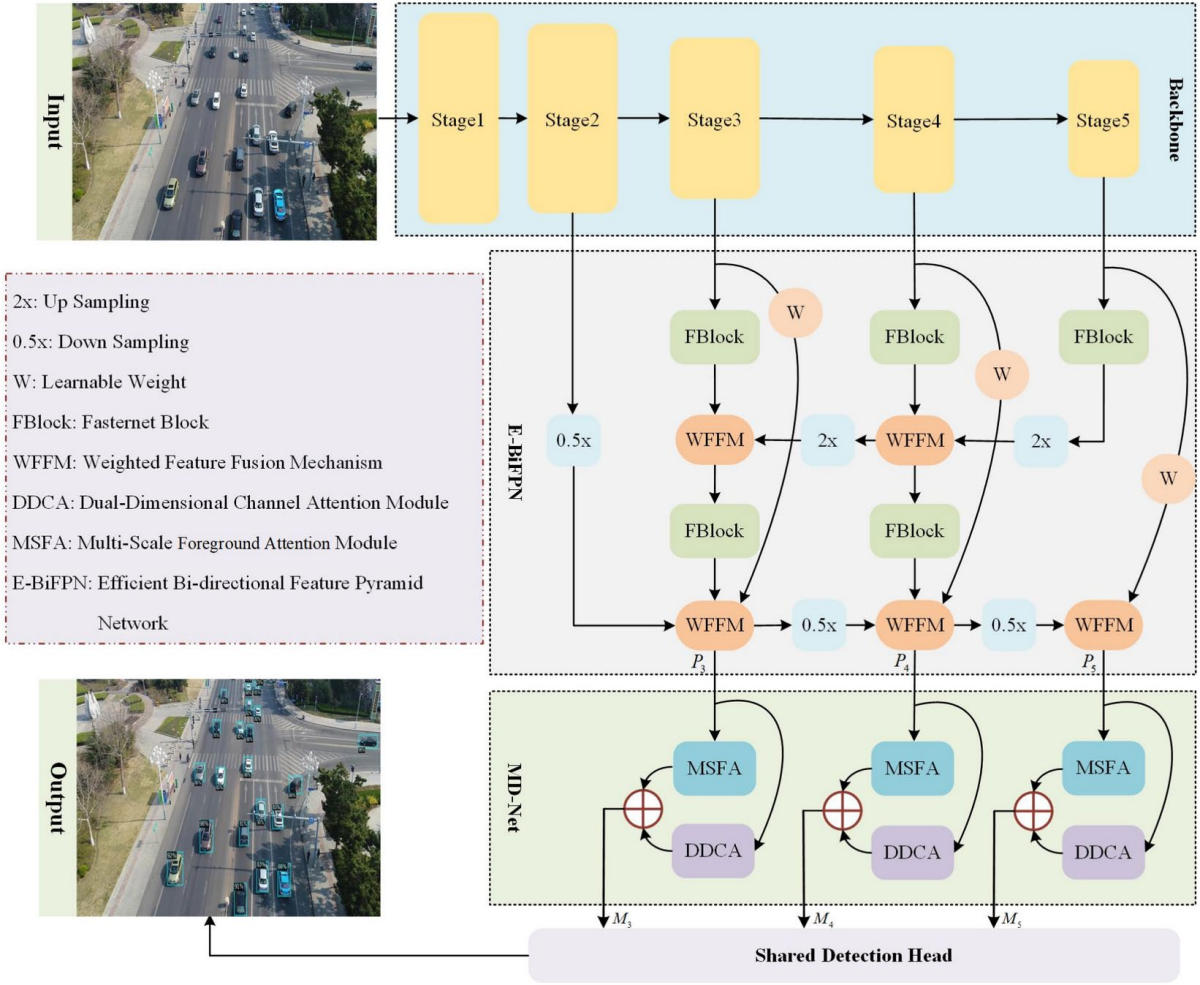
The framework of the proposed Collaborative Multi-Attention Network (CMA-Net), featuring the ResNet-50 backbone, E-BiFPN for feature fusion, DM-Net for collaborative attention, and Shared detection head.

The overall architecture of the proposed Collaborative Multi-Attention Network (CMA-Net), designed for real-time UAV small object detection, is illustrated in Figure 1. The framework consists of four principal components. First, ResNet-50 is employed as the backbone network owing to its effective balance between accuracy and computational efficiency. Subsequently, an Efficient Bi-directional Feature Pyramid Network (E-BiFPN) is utilized to achieve multi-scale weighted feature fusion, enhancing the representation of small objects while minimizing computational overhead. Following this, the Dual-Dimensional Channel Attention (DDCA) and Multi-Scale Foreground Attention (MSFA) modules are integrated in parallel to form a unified DM-Net, which collaboratively captures inter-object relationships across different scales and inter-channel correlations across dimensions, thereby further strengthening feature representation for small objects. Finally, an anchor-free share detection head is adopted to perform accurate regression and classification, eliminating the need for predefined anchor boxes and reducing computational complexity, which collectively improves both efficiency and detection performance.

### Efficient Bi-directional feature pyramid network

Built upon the ResNet-50 backbone, we construct an Efficient Bi-directional Feature Pyramid Network (E-BiFPN) that utilizes features from Stage2 to Stage5 to generate multi-scale feature layers $P_{3}$-$P_{5}$. Specifically designed for small object detection, our architecture omits the higher-level P6/P7 layers employed in standard Bi-FPN^13^, while introducing an additional down-sampling path from Stage 2 to enhance the representation of detailed spatial features critical for small targets.

**Fig. 2:**
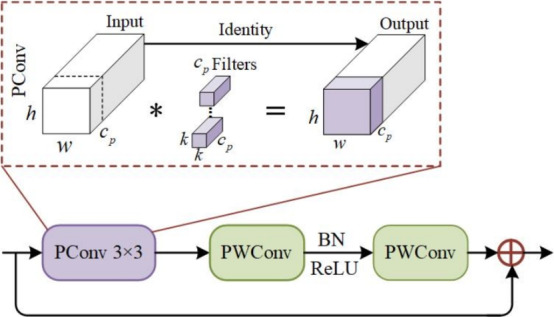
The structure diagram of FBlock.

To optimize computational efficiency, we integrate the Fasternet Block^41^ (denoted as FBlock in Figure 1) throughout the E-BiFPN structure, with its detailed architecture presented in Figure 2. The FBlock architecture comprises a partial convolution (PConv) module followed by two pointwise convolution (PWConv) layers. The PConv layer performs convolution on a subset of input channels using $c_{p}$ filters, each of size $k \,\times \, k \,\times \,c_{p}$, and concatenates the output with the remaining unchanged channels. Subsequently, the first PWConv layer expands the channel dimensionality while incorporating activation functions to enhance nonlinear representation capacity. The second PWConv layer then compresses the channel dimension to retain the most salient features. This design greatly reduces computational complexity while maintaining high accuracy. Furthermore, the E-BiFPN incorporates a Weighted Feature Fusion Mechanism (WFFM) that adaptively learns scale-specific weights to improve fusion effectiveness. This mechanism alleviates the inherent limitation where small object features tend to diminish in deeper layers while interference from background information persists. By adaptively increasing the contribution of shallow features and suppressing that of deeper ones, WFFM enhances the representation of small targets while reducing background interference. The WFFM operation multiplies feature maps of identical resolution by their learnable weights before summation. Initialized to unity, these weights are automatically optimized during training through gradient descent. This approach not only improves fusion effectiveness but also reduces parameter count.

The bidirectional architecture of E-BiFPN enables complementary feature enhancement: the top-down pathway enriches shallow features with high-level semantic context, while the bottom-up pathway augments deep features with spatial details from shallow layers. This synergistic multi-scale integration enhances feature representation while reducing computational complexity, making it particularly suitable for accurate real-time detection of small objects in UAV imagery.

### Dual-dimensional channel attention module

The DDCA module adaptively recalibrates channel-wise feature responses by operating along both the width and height dimensions of feature maps. Unlike conventional channel attention mechanisms that rely on single-dimensional compression, DDCA module employs separate global average pooling operations along the horizontal and vertical axes, enabling it to capture inter-channel dependencies while preserving precise spatial information. This dual-dimensional design allows the module to focus on long-range dependencies in both spatial directions, effectively suppressing redundant channels in the feature pyramid network while enhancing spatial sensitivity. By emphasizing crucial channels and strengthening discriminative features, DDCA module significantly improves the representation and detection accuracy of small and dim objects.

**Fig. 3:**
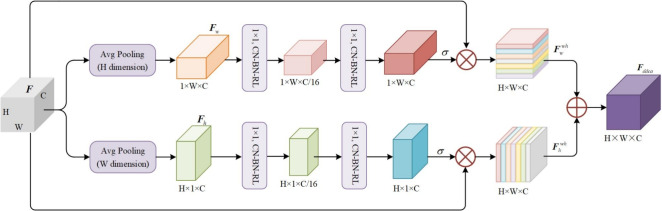
The structure for dual-dimensional channel attention module (DDCA).

The overall structure of the DDCA Module, illustrated in Figure 3, primarily consists of two parallel branches: the width-direction branch and the height-direction branch. Specifically, in the width-direction branch, the input feature map *F* undergoes global average pooling along the height (H) dimension to produce the width-direction feature map $F_{w}$, which captures significant features from the left and right regions of the image. This feature map $F_{w}$ is then processed through a series of operations including convolution, batch normalization (BN), and non-linear activation functions to generate the width attention weights. Finally, the width attention weights are multiplied channel-wise with the original input feature *F* to obtain the horizontally enhanced feature $F_{w}^{wh}$. This process can be represented by Equation (1):

1 $$\begin{aligned} F_{w}^{wh} = F \times {\sigma (Conv_2(Conv_1(AVG_{H}(F))))} \end{aligned}$$

where $AVG_{H}(\cdot )$ denotes global average pooling along the H dimension. $Conv_1(\cdot )$ refers to a 1$\,\times \,$1 convolutional layer followed by BN and ReLU activation, which reduces the channel number of $F_{w}$ to 1/16 of the original. $Conv_2(\cdot )$ represents another 1$\,\times \,$1 convolutional layer with BN and ReLU activation that restores the channel number to match the original feature map. The inclusion of $Conv_1(\cdot )$ and $Conv_2(\cdot )$ aims to recalibrate the feature channels along the width dimension, enhancing the model’s focus on important features and its representational capacity, thereby improving generalization. $\sigma (\cdot )$ represents using the sigmoid activation function to obtain width attention weights from the output of $Conv_2(\cdot )$.

Similarly, in the height-direction branch, the input feature map *F* undergoes global average pooling along the width (W) dimension to produce the height-direction feature map $F_{h}$, which captures significant features from the upper and lower regions of the image. This feature map $F_{h}$ is then processed through corresponding convolution, BN, and non-linear activation operations to generate the height attention weights. Finally, these height attention weights are multiplied channel-wise with the original input feature *F* to obtain the vertically enhanced feature $F_{h}^{wh}$. This procedure can be formulated as Equation (2):

2 $$\begin{aligned} F_{h}^{wh} = F \times {\sigma (Conv_2(Conv_1(AVG_{W}(F))))} \end{aligned}$$

where $AVG_{W}(\cdot )$ denotes global average pooling along the W dimension. The inclusion of $Conv_1(\cdot )$ and $Conv_2(\cdot )$ serves to reevaluate and recalibrate the feature channels along the height dimension, enabling the model to better focus on crucial features while suppressing less important ones. $\sigma (\cdot )$ represents using the sigmoid activation function to obtain height attention weights from the output of $Conv_2(\cdot )$.

Finally, the horizontally enhanced feature $F_{w}^{wh}$ and the vertically enhanced feature $F_{h}^{wh}$ are fused through element-wise summation to form a more discriminative feature representation $F_{ddca}$. In summary, the DDCA module enables feature maps in each channel to be weighted according to their spatial importance along both horizontal and vertical directions. This mechanism enhances the model’s focus on semantically significant features at specific spatial locations, thereby significantly improving detection performance for small objects in complex backgrounds.

### Multi-scale foreground attention module

The Multi-Scale Foreground Attention (MSFA) module is a distinctive foreground attention mechanism that establishes connections between large objects with high-level semantic information in deep layers and small objects with detailed features in shallow layers. Unlike conventional attention mechanisms that only capture single-scale correlations within the same feature layer, the MSFA module uniformly samples and fuses the three-scale features from E-BiFPN along the channel dimension, enabling it to explore correlations across different scales. This design enhances the model’s focus on small objects while suppressing irrelevant background interference, thereby significantly improving the representation of small objects and the model’s discriminative power.

**Fig. 4:**
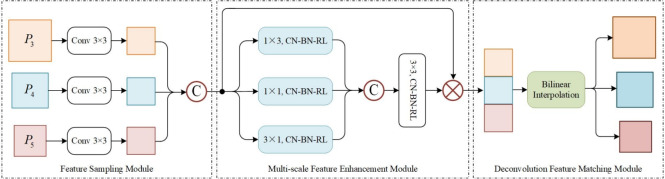
The structure for multi-scale foreground attention module (MSFA).

The overall architecture of the MSFA module, illustrated in Figure 4, consists of three key components: a feature sampling module, a multi-scale feature enhancement module, and a deconvolution feature matching module. Specifically, the feature sampling module first processes the three-level features $P_{3}$, $P_{4}$, $P_{5}$ from E-BiFPN using 3$\,\times \,$3 convolutional kernels to unify their spatial resolutions, then concatenates the resampled features to establish connections between objects across different scales. However, this concatenation operation inevitably introduces background irrelevant to small object detection. To address this, the multi-scale feature enhancement module is employed to focus on foreground features while suppressing background interference and improving computational efficiency. This enhancement module extracts multi-scale features using parallel convolutions with kernel sizes of 1$\,\times \,$3, 1$\,\times \,$1, and 3$\,\times \,$1, concatenates them along the channel dimension to enhance nonlinear representation capability, and then applies 3$\,\times \,$3 convolutional operations to capture spatial dependencies and generate foreground attention weights. These weights are subsequently multiplied channel-wise with the input features to produce enhanced foreground features. Unlike conventional channel attention methods that rely on global pooling operations, this specialized foreground attention mechanism effectively enhances multi-scale foreground representation. In the deconvolution feature matching module, bilinear interpolation is applied to restore the enhanced foreground features to the original scales of E-BiFPN’s three prediction layers, enabling effective feature matching with DDCA module outputs while preserving spatial information.

**Fig. 5:**
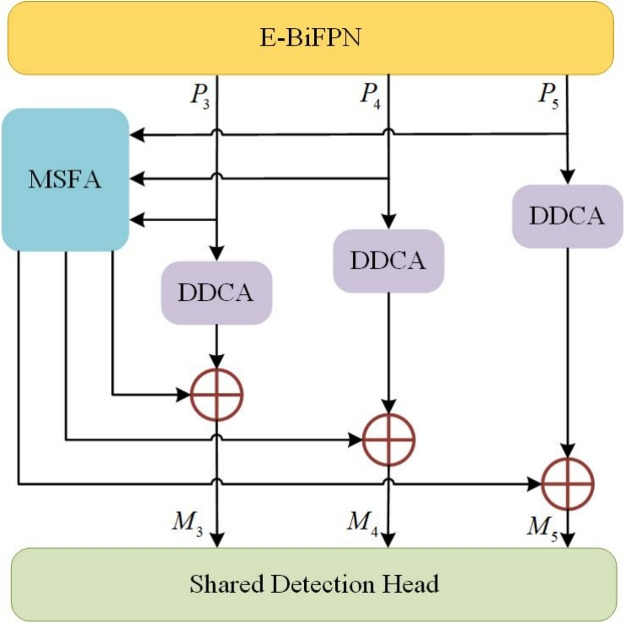
Schematic illustration of the collaborative dataflow among the E-BiFPN, DDCA, and MSFA modules.

Finally, Figure 5 illustrates the specific relationships and collaborative dataflow among the three core components: E-BiFPN, DDCA, and MSFA. Multi-scale features from the backbone are first enhanced and fused by the E-BiFPN module, producing outputs denoted as P3, P4, and P5. These features are then processed in parallel by two specialized branches: the DDCA branch, which captures inter-channel correlations across dimensions, and the MSFA branch, which models inter-object relationships across different feature levels. The outputs from these two paths are fused via element-wise addition, forming comprehensive representations M3, M4, and M5, which are finally passed to the detection head. This design highlights the complementary and synergistic integration of channel refinement and cross-scale spatial aggregation in our model.

### Shared detection head

**Fig. 6:**
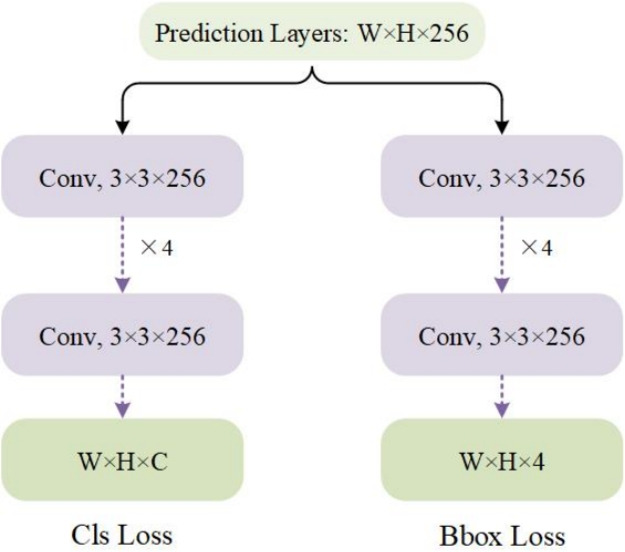
The structure for shared detection head.

The shared detection head employs an anchor-free mechanism^42^ for small object regression and classification, with its detailed architecture illustrated in Figure 6. The proposed design separates the detection head into two parallel branches: a classification branch and a regression branch. Both branches sequentially apply four 3$\,\times \,$3 convolutional layers to each prediction layer from the MD-Net. The classification branch predicts object categories, while the regression branch estimates bounding box locations. All parameters in the detection head are shared across different prediction layers. For training, we employ separate loss functions for classification and regression tasks. The classification loss combines Focal Loss^24^ and Cross Entropy Loss^7^ to address class imbalance, while the regression loss utilizes GIoU Loss^43^ for accurate bounding box prediction. This design enables simultaneous prediction of a *C*-dimensional class vector (*C* represents the number of categories) and a 4-dimensional bounding box vector, achieving efficient and accurate small object detection.

## Experiments and analysis

### Datasets and evaluation metrics

Our method is evaluated on two challenging datasets: UAVDT and Stanford Drone, both characterized by multi-scenario and strong interference conditions. UAVDT dataset comprises 39,850 images, with 23,258 for training and 16,592 for testing. All images have a resolution of approximately 1080$\,\times \,$540 pixels. Stanford Drone dataset consists of 3,500 training images and 831 test images, captured at a resolution of approximately 1409$\,\times \,$1916 pixels. We evaluate our method using standard MS COCO metrics, including $\text {AP}$, $\text {AP}_{50}$, $\text {AP}_{75}$, $\text {AP}_S$, $\text {AP}_M$, and $\text {AP}_L$. Specifically, Average Precision (AP) represents the mean accuracy across all categories at 10 Intersection over Union (IoU) thresholds sampled from 0.5 to 0.95 with an interval of 0.05. $\text {AP}_{50}$ and $\text {AP}_{75}$ denote the average precision when the IoU between predictions and ground truth exceeds 0.5 and 0.75, respectively. $\text {AP}_S$, $\text {AP}_M$, and $\text {AP}_L$ measure accuracy for small (area $<{32^2}$), medium ($32^2<$ area $<96^2$), and large (area $\>96^2$) objects, respectively. Additionally, we employed Frames Per Second (FPS) to assess the detection speed of our model.

### Implementation details

All experiments were implemented on an Ubuntu 22.04 operating system using the PyTorch deep learning framework. The configuration employed CUDA 11.7, PyTorch 1.13.1, cuDNN 8.5.0, and Python 3.8. The backbone network was initialized with pre-trained weights from ImageNet. The model was trained for 100k iterations with an initial learning rate of 0.01, which was reduced to 0.001 and 0.0001 at the 60k and 80k iterations, respectively. The input images were resized to 600$\,\times \,$600 pixels, with a batch size of 8 for training.

**Table 1:** Results of different detection methods for UAVDT dataset.

Methods	Backbone	Input Size	$\text {AP}$	$\text {AP}_{50}$	$\text {AP}_{75}$	$\text {AP}_S$	$\text {AP}_M$	$\text {AP}_L$	FPS
Two-stage detectors									
FPN^12^	ResNet-50	900$\,\times \,$600	56.8	87.5	64.6	43.6	72.2	80.2	16
Retinanet^24^	ResNet-50	900$\,\times \,$600	47.0	74.9	51.9	22.7	72.2	81.7	19
Mask R-CNN^44^	ResNet-50	900$\,\times \,$600	57.1	87.4	65.0	44.1	72.6	79.7	17
End-to-end detectors									
DINO-Deformable DETR^45^	ResNet-50	1333$\,\times \,$800	20.5	34.4	21.8	15.4	32.7	37.0	5
Deformable DETR^46^	ResNet-50	1333$\,\times \,$800	17.4	29.3	18.9	12.1	29.4	32.5	19
RT-DETR^47^	ResNet-50	640$\,\times \,$640	24.2	39.1	26.7	17.4	36.3	41.0	108
Single-stage detectors									
YOLOv7^9^	CSPDarknet-53	640$\,\times \,$640	17.3	32.4	-	-	-	-	70
YOLOv8^10^	CSPDarknet-53	640$\,\times \,$640	20.5	34.0	-	-	-	-	107
YOLOv10^11^	CSPNet	640$\,\times \,$640	19.5	32.1	-	-	-	-	130
YOLOv11^48^	CSPNet	640$\,\times \,$640	18.4	30.6	-	-	-	-	143
MAFDet^49^	MS-Swin transformer	800$\,\times \,$800	21.6	35.5	24.4	15.9	33.2	26.3	38
LWUAVDet^50^	CSPDarknet-53	640$\,\times \,$640	18.3	34.1	-	-	-	-	60
ASCDet^51^	ResNet-50	1024$\,\times \,$540	18.0	31.8	18.8	-	-	-	27
FCOS^42^	ResNet-50	900$\,\times \,$600	64.8	**95.9**	74.9	53.1	77.6	88.1	35
CMA-Net	MobilenetV3	600$\,\times \,$600	61.1	90.5	69.5	48.1	67.6	85.8	75
CMA-Net	EfficientNet-Lite4	600$\,\times \,$600	65.2	93.2	73.8	52.3	75.7	90.1	68
CMA-Net (our)	ResNet-50	600$\,\times \,$600	**67.2**	95.8	**78.3**	**54.7**	**81.3**	**93.7**	64

### Overall performance

#### Comparative analysis of overall performance on UAVDT dataset

On the UAVDT dataset, we evaluate the proposed method against several state-of-the-art detectors using standard metrics including $\text {AP}$, $\text {AP}_{50}$, $\text {AP}_{75}$, $\text {AP}_S$, $\text {AP}_M$, and $\text {AP}_L$, and FPS. As summarized in Table 1, our approach outperforms classical two-stage detectors such as FPN^12^, RetinaNet^24^, and Mask R-CNN^44^, achieving AP improvements of 10.4%, 20.2%, and 10.1%, respectively. These gains are attributed to the synergistic effect of the E-BiFPN, DDCA, and MSFA modules, which enhance discriminative capability for small objects while suppressing redundant information and maintaining a real-time inference speed of 64 FPS. Furthermore, when compared with leading end-to-end detection frameworks including DINO-Deformable DETR^45^, Deformable DETR^46^, and RT-DETR^47^, our method achieves superior performance across most evaluation metrics, as shown in Table 1. Although RT-DETR exhibits faster inference speed, our approach excels in overall detection accuracy. Finally, compared to efficient one-stage detectors such as the YOLO series^9–11,48^ and other methods^42,49–51^, our method delivers the best performance in terms of $\text {AP}$ and $\text {AP}_{75}$, despite a lower FPS. Notably, our detector shows particularly strong performance on small objects (area $<{32^2}$), striking an effective balance between real-time capability and detection accuracy.

#### Comparative analysis of overall performance on stanford drone dataset

On the Stanford Drone dataset, we further evaluated the proposed method using standard metrics including $\text {AP}$, $\text {AP}_{50}$, $\text {AP}_{75}$, $\text {AP}_S$, $\text {AP}_M$, and $\text {AP}_L$, and FPS to validate its practical utility and effectiveness. As summarized in Table 2, comprehensive comparisons were conducted against three categories of detectors: high-accuracy two-stage methods, state-of-the-art end-to-end approaches, and efficient single-stage models. Experimental results demonstrate that our method achieves 62.0% $\text {AP}$ with 64 FPS on this dataset, successfully balancing accuracy and speed for real-time small object detection in UAV imagery.

**Table 2:** Results of different detection methods for Stanford Drone dataset.

Methods	Backbone	Input Size	$\text {AP}$	$\text {AP}_{50}$	$\text {AP}_{75}$	$\text {AP}_S$	$\text {AP}_M$	$\text {AP}_L$	FPS
Two-stage detectors									
FPN^12^	ResNet-50	900$\,\times \,$600	29.3	56.5	26.0	10.8	25.9	37.6	16
Retinanet^24^	ResNet-50	900$\,\times \,$600	29.8	54.4	27.2	11.3	25.7	41.1	19
Mask R-CNN^44^	ResNet-50	900$\,\times \,$600	29.3	55.0	27.4	12.3	26.0	37.2	17
End-to-end detectors									
DINO-Deformable DETR^45^	ResNet-50	1333$\,\times \,$800	40.3	72.0	40.5	13.0	35.9	50.3	5
Deformable DETR^46^	ResNet-50	1333$\,\times \,$800	29.6	64.5	23.2	7.0	27.6	33.3	19
RT-DETR^47^	ResNet-50	640$\,\times \,$640	44.5	74.4	48.2	**20.7**	41.0	51.5	108
Single-stage detectors									
YOLOv7^9^	CSPDarknet-53	640$\,\times \,$640	28.9	57.0	-	-	-	-	70
YOLOv8^10^	CSPDarknet-53	640$\,\times \,$640	42.1	71.1	-	-	-	-	107
YOLOv10^11^	CSPNet	640$\,\times \,$640	41.7	70.5	-	-	-	-	130
YOLOv11^48^	CSPNet	640$\,\times \,$640	55.0	77.6	-	-	-	-	143
MAFDet^49^	MS-Swin transformer	800$\,\times \,$800	53.9	76.1	49.8	12.5	40.4	53.9	38
LWUAVDet^50^	CSPDarknet-53	640$\,\times \,$640	47.0	75.2	-	-	-	-	60
ASCDet^51^	ResNet-50	1024$\,\times \,$540	45.0	72.3	18.8	-	-	-	27
FCOS^42^	ResNet-50	900$\,\times \,$600	53.6	81.2	58.8	15.6	50.0	65.1	35
CMA-Net (our)	ResNet-50	600$\,\times \,$600	**62.0**	**82.2**	**67.2**	16.2	**58.0**	**74.9**	64

This performance can be primarily attributed to the effective collaboration between our attention modules: the MSFA enhances feature representation for small objects and improves recall rates, while the DDCA suppresses background interference and reduces false detections. These consistent results across different datasets confirm the strong practicality and generalization capability of our CMA-Net for real-time small object detection.

### Ablation studies

Ablation studies on UAVDT and Stanford Drone datasets evaluate the contributions of key CMA-Net components, including E-BiFPN, DDCA, and MSFA, under identical experimental settings.

#### The effectiveness of E-BiFPN

To validate the effectiveness of the proposed E-BiFPN, we conducted comparative experiments with four distinct model configurations: a baseline ResNet-50 without feature fusion, ResNet-50 with FPN for unidirectional semantic enhancement, ResNet-50 with Bi-FPN to address feature imbalance, and ResNet-50 with our E-BiFPN for bidirectional feature guidance. As summarized in Table 3, E-BiFPN demonstrates significant improvement in AP metrics, achieving 65.7% on UAVDT and 58.1% on Stanford Drone datasets.

**Table 3:** Performance comparison of E-BiFPN module with different fusion modules on UAVDT and Stanford Drone datasets.

Datasets	Methods	$\text {AP}$	$\text {AP}_{50}$	$\text {AP}_{75}$	$\text {AP}_S$	$\text {AP}_M$	$\text {AP}_L$	Params(M)	GFLOPs
UAVDT	ResNet-50	62.5	92.8	72.1	49.1	77.4	89.3	25.9	73.2
	+ FPN	64.1($\uparrow$1.6)	93.3($\uparrow$0.5)	73.1($\uparrow$1.0)	50.0($\uparrow$0.9)	77.6($\uparrow$0.2)	90.3($\uparrow$1.0)	28.4	95.6
	+ Bi-FPN	65.2($\uparrow$2.7)	94.1($\uparrow$1.3)	75.7($\uparrow$3.6)	51.5($\uparrow$2.4)	80.2($\uparrow$2.8)	**92.7(**$\uparrow$**3.4)**	31.1	118.6
	+ E-BiFPN	**65.7(**$\uparrow$**3.2)**	**94.1(**$\uparrow$**1.3)**	**76.4(**$\uparrow$**4.3)**	**52.7(**$\uparrow$**3.6)**	**80.5(**$\uparrow$**3.1)**	92.5($\uparrow$3.2)	27.4	87.1
Stanford Drone	ResNet-50	41.2	69.7	43.7	12.9	36.1	48.3	25.9	73.2
	+ FPN	45.6($\uparrow$4.4)	72.2($\uparrow$2.5)	49.8($\uparrow$6.1)	13.5($\uparrow$0.6)	40.7($\uparrow$4.6)	54.9($\uparrow$6.6)	28.4	95.6
	+ Bi-FPN	57.0($\uparrow$15.8)	79.3($\uparrow$9.6)	60.9($\uparrow$17.2)	14.9($\uparrow$2.0)	53.0($\uparrow$16.9)	**70.3(**$\uparrow$**22.0)**	31.1	118.6
	+ E-BiFPN	**58.1(**$\uparrow$**16.9)**	**81.2(**$\uparrow$**11.5)**	**63.0(**$\uparrow$**19.3)**	**15.3(**$\uparrow$**2.4)**	**54.9(**$\uparrow$**18.8)**	70.0($\uparrow$21.7)	27.4	87.1

$\uparrow$ denotes an improvement over the baseline, $\downarrow$ indicates a degradation, and ”-” represents no change in performance

Notably, for small object detection ($\text {AP}_S$), E-BiFPN shows gains of 3.6%, 2.7%, and 1.2% over the other three models on UAVDT, and improvements of 2.4%, 1.8%, and 0.4% on Stanford Drone dataset, respectively. Furthermore, compared to FPN and Bi-FPN, the integration of FBlock and WFFM not only enhances detection performance but also reduces both GFLOPs and parameter count. These results confirm that E-BiFPN effectively mitigates feature loss in small objects by balancing the fusion of shallow details and deep semantics, thereby improving detection accuracy while maintaining a parameter-efficient structure.

#### The importance of DDCA

**Fig. 7:**
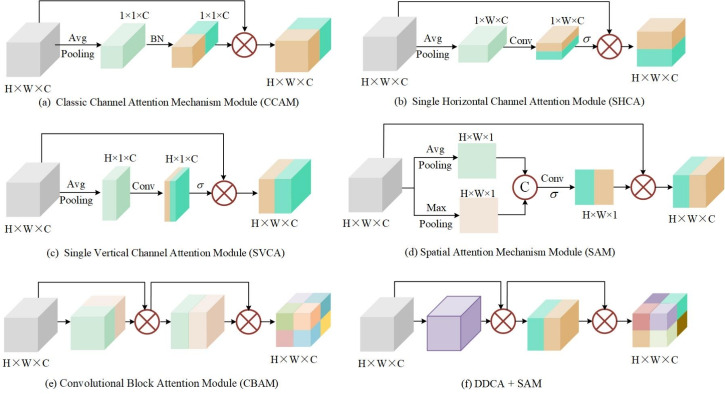
The structure for different attention mechanism modules.

Building upon the E-BiFPN architecture, we systematically evaluate the contribution of DDCA module through comprehensive comparisons with established attention mechanisms. As illustrated in Figure 7, the compared modules include: Classic Channel Attention Mechanism (CCAM) Module^34^ (Figure 7 (a)), Single Horizontal Channel Attention (SHCA) Module (Figure 7 (b)), Single Vertical Channel Attention (SVCA) Module (Figure 7 (c)), Spatial Attention Mechanism (SAM) Module^35^ (Figure 7 (d)), Convolutional Block Attention Module (CBAM)^35^ (Figure 7 (e)), and a hybrid DDCA+SAM configuration (Figure 7 (f)).

**Table 4:** Performance comparison of DDCA with different attention modules on UAVDT and Stanford Drone datasets.

Datasets	Methods	$\text {AP}$	$\text {AP}_{50}$	$\text {AP}_{75}$	$\text {AP}_S$	$\text {AP}_M$	$\text {AP}_L$
UAVDT	E-BiFPN	65.7	94.1	76.4	52.7	80.5	92.5
	+ CCAM	66.2($\uparrow$0.5)	94.6($\uparrow$0.5)	76.6($\uparrow$0.2)	53.2($\uparrow$0.5)	80.8($\uparrow$0.3)	92.9($\uparrow$0.4)
	+ SHCA	66.0($\uparrow$0.3)	94.5($\uparrow$0.4)	76.6($\uparrow$0.2)	53.0($\uparrow$0.3)	80.7($\uparrow$0.2)	92.6($\uparrow$0.1)
	+ SVCA	65.9($\uparrow$0.2)	94.1(-)	76.8($\uparrow$0.4)	53.1($\uparrow$0.4)	80.5(-)	92.5(-)
	+ SAM	66.2($\uparrow$0.5)	94.5($\uparrow$0.4)	76.8($\uparrow$0.4)	53.2($\uparrow$0.5)	80.8($\uparrow$0.3)	92.6($\uparrow$0.1)
	+ CBAM	66.4($\uparrow$0.7)	94.4($\uparrow$0.3)	77.1($\uparrow$0.7)	53.4($\uparrow$0.7)	81.0($\uparrow$0.5)	**93.3(** $\uparrow$ **0.8)**
	+ DDCA+SAM	66.3($\uparrow$0.6)	94.4($\uparrow$0.3)	77.0($\uparrow$0.6)	53.3($\uparrow$0.6)	80.9($\uparrow$0.4)	**93.3(** $\uparrow$ **0.8)**
	+ DDCA	**66.5(** $\uparrow$ **0.8)**	**94.6(** $\uparrow$ **0.5)**	**77.2(** $\uparrow$ **0.8)**	**53.7(** $\uparrow$ **1.0)**	**81.1(** $\uparrow$ **0.6)**	93.2($\uparrow$0.7)
Stanford Drone	E-BiFPN	58.1	81.2	63.0	15.3	54.9	70.0
	+ CCAM	59.1($\uparrow$1.0)	80.6($\downarrow$0.6)	63.7($\uparrow$0.7)	14.6($\downarrow$0.7)	55.8($\uparrow$0.9)	71.1($\uparrow$1.1)
	+ SHCA	58.4($\uparrow$0.3)	81.1($\downarrow$0.1)	63.1($\uparrow$0.1)	14.3($\downarrow$1.0)	55.4($\uparrow$0.5)	70.3($\uparrow$0.3)
	+ SVCA	58.1(-)	80.1($\downarrow$1.1)	63.0(-)	14.3($\downarrow$1.0)	54.9(-)	70.0(-)
	+ SAM	58.7($\uparrow$0.6)	80.6($\downarrow$0.6)	63.7($\uparrow$0.7)	14.6($\downarrow$0.7)	55.3($\uparrow$0.4)	70.9($\uparrow$0.9)
	+ CBAM	59.4($\uparrow$1.3)	80.1($\downarrow$1.1)	63.9($\uparrow$0.9)	14.9($\downarrow$0.4)	55.7($\uparrow$0.8)	72.0($\uparrow$2.0)
	+ DDCA+SAM	60.2($\uparrow$2.1)	81.4($\uparrow$0.2)	64.8($\uparrow$1.8)	15.1($\downarrow$0.2)	55.6($\uparrow$0.7)	73.2($\uparrow$3.2)
	+ DDCA	**61.7(** $\uparrow$ **3.6)**	**82.2(** $\uparrow$ **1.0)**	**65.8(** $\uparrow$ **2.8)**	15.2($\downarrow$0.1)	**57.0(** $\uparrow$ **2.1)**	**73.5(** $\uparrow$ **3.5)**

As quantified in Table 4, DDCA demonstrates consistent improvements over unidirectional channel attention mechanisms. On UAVDT dataset, it achieves $\text {AP}$ gains of 0.3%, 0.5%, and 0.6% compared to CCAM, SHCA, and SVCA, respectively. These improvements are more pronounced on Stanford Drone dataset, with corresponding gains of 2.6%, 3.3%, and 3.6%. This is because DDCA can adaptively recalibrates feature channel weights across both horizontal and vertical spatial axes, while modeling correlations among critical channels. When compared to the SAM, DDCA maintains superior performance with an $\text {AP}$ improvement of 0.3% on UAVDT dataset and 3.0% on Stanford Drone dataset, alongside $\text {AP}_S$ enhancements of 0.5% and 0.6% respectively. This demonstrates that DDCA not only emphasizes important channels but also leverages cross-dimensional correlations to focus on spatially significant regions. Notably, DDCA outperforms both hybrid attention mechanisms including CBAM and the combined DDCA plus SAM configuration. This is because some certain features may be activated in the bidirectional channel dimension but suppressed in the spatial dimension, thereby masking some additional important information and resulting in a decrease in the performance of the hybrid attention mechanism. The experimental results conclusively demonstrate that DDCA significantly enhances small object detection accuracy through its coordinated dual-dimensional feature recalibration.

**Table 5:** Performance comparison of MSFA module on UAVDT and Stanford Drone datasets.

Datasets	E-BiFPN	SSM	TSM	MSFA	DDCA	$\text {AP}$	$\text {AP}_{50}$	$\text {AP}_{75}$	$\text {AP}_S$	$\text {AP}_M$	$\text {AP}_L$
UAVDT	$\checkmark$					65.7	94.1	76.4	52.7	80.5	92.5
	$\checkmark$	$\checkmark$				66.0($\uparrow$0.3)	94.5($\uparrow$0.4)	77.1($\uparrow$0.7)	53.1($\uparrow$0.4)	80.5(-)	93.0($\uparrow$0.5)
	$\checkmark$		$\checkmark$			66.3($\uparrow$0.6)	94.1(-)	77.3($\uparrow$0.9)	53.5($\uparrow$0.8)	80.9($\uparrow$0.4)	93.1($\uparrow$0.6)
	$\checkmark$			$\checkmark$		66.6($\uparrow$0.9)	94.4($\uparrow$0.3)	78.0($\uparrow$1.6)	54.2($\uparrow$1.5)	80.5(-)	92.9($\uparrow$0.4)
	$\checkmark$	$\checkmark$			$\checkmark$	66.5($\uparrow$0.8)	94.7($\uparrow$0.6)	77.3($\uparrow$0.9)	53.5($\uparrow$0.8)	81.1($\uparrow$0.6)	93.6($\uparrow$1.1)
	$\checkmark$		$\checkmark$		$\checkmark$	66.8($\uparrow$1.1)	94.7($\uparrow$0.6)	78.0($\uparrow$1.6)	54.2($\uparrow$1.5)	81.0($\uparrow$0.5)	93.5($\uparrow$1.0)
	$\checkmark$			$\checkmark$	$\checkmark$	**67.2(**$\uparrow$**1.5)**	**95.8(**$\uparrow$**1.7)**	**78.3(**$\uparrow$**1.9)**	**54.7(**$\uparrow$**2.0)**	**81.3(**$\uparrow$**0.8)**	**93.7(**$\uparrow$**1.2)**
Stanford Drone	$\checkmark$					58.1	81.2	63.0	15.3	54.9	70.0
	$\checkmark$	$\checkmark$				58.6($\uparrow$0.5)	80.6($\downarrow$0.6)	64.0($\uparrow$1.0)	15.4($\uparrow$0.1)	55.2($\uparrow$0.3)	70.8($\uparrow$0.8)
	$\checkmark$		$\checkmark$			59.2($\uparrow$1.1)	81.3($\uparrow$0.1)	64.4($\uparrow$1.4)	15.3(-)	55.6($\uparrow$0.7)	70.8($\uparrow$0.8)
	$\checkmark$			$\checkmark$		61.2($\uparrow$3.1)	80.9($\downarrow$0.3)	66.3($\uparrow$3.3)	15.7($\uparrow$0.4)	57.5($\uparrow$2.6)	74.6($\uparrow$4.6)
	$\checkmark$	$\checkmark$			$\checkmark$	61.1($\uparrow$3.0)	81.2(-)	64.1($\uparrow$1.1)	15.5($\uparrow$0.2)	57.4($\uparrow$2.5)	73.9($\uparrow$3.9)
	$\checkmark$		$\checkmark$		$\checkmark$	61.5($\uparrow$3.4)	80.8($\downarrow$0.4)	66.7($\uparrow$3.7)	15.6($\uparrow$0.3)	57.4($\uparrow$2.5)	74.8($\uparrow$4.8)
	$\checkmark$			$\checkmark$	$\checkmark$	**62.0(**$\uparrow$**3.9)**	**82.2(**$\uparrow$**1.0)**	**67.2(**$\uparrow$**4.2)**	**16.2(**$\uparrow$**0.9)**	**58.0(**$\uparrow$**3.1)**	**74.9(**$\uparrow$**4.9)**

#### The necessity of MSFA

As demonstrated in Table 5 through comparisons of rows 2 and 5 with rows 9 and 12, the MSFA module improves $\text {AP}$ by 0.9% on UAVDT and 3.1% on Stanford Drone datasets with corresponding $\text {AP}_S$ gains of 1.5% and 0.4%, demonstrating its critical importance to model performance. To further validate the effectiveness of the MSFA design, we replace its original multi-scale feature enhancement module, which employs 1$\,\times \,$3, 1$\,\times \,$1, and 3$\,\times \,$1 kernels, with two alternative configurations: a Two-Scale Module termed TSM using 1$\,\times \,$1 and 3$\,\times \,$3 kernels, and a Single-Scale Module termed SSM using only 3$\,\times \,$3 kernel, while maintaining all other settings unchanged. Comparative results from rows 3 to 5 in Table 5 on UAVDT dataset indicate that multi-scale designs surpass single-scale modules, a finding we attribute to their richer receptive fields that enable more comprehensive feature extraction. Furthermore, a comparison between rows 4 and 5 shows that features extracted using combined 1$\,\times \,$3 and 3$\,\times \,$1 kernels yield more refined details than those obtained with a single 3$\,\times \,$3 kernel, thereby enhancing small object representation. This improvement, however, comes with a slight reduction in $\text {AP}_L$ for large objects, likely resulting from the increased emphasis on detailed patterns.

**Fig. 8:**
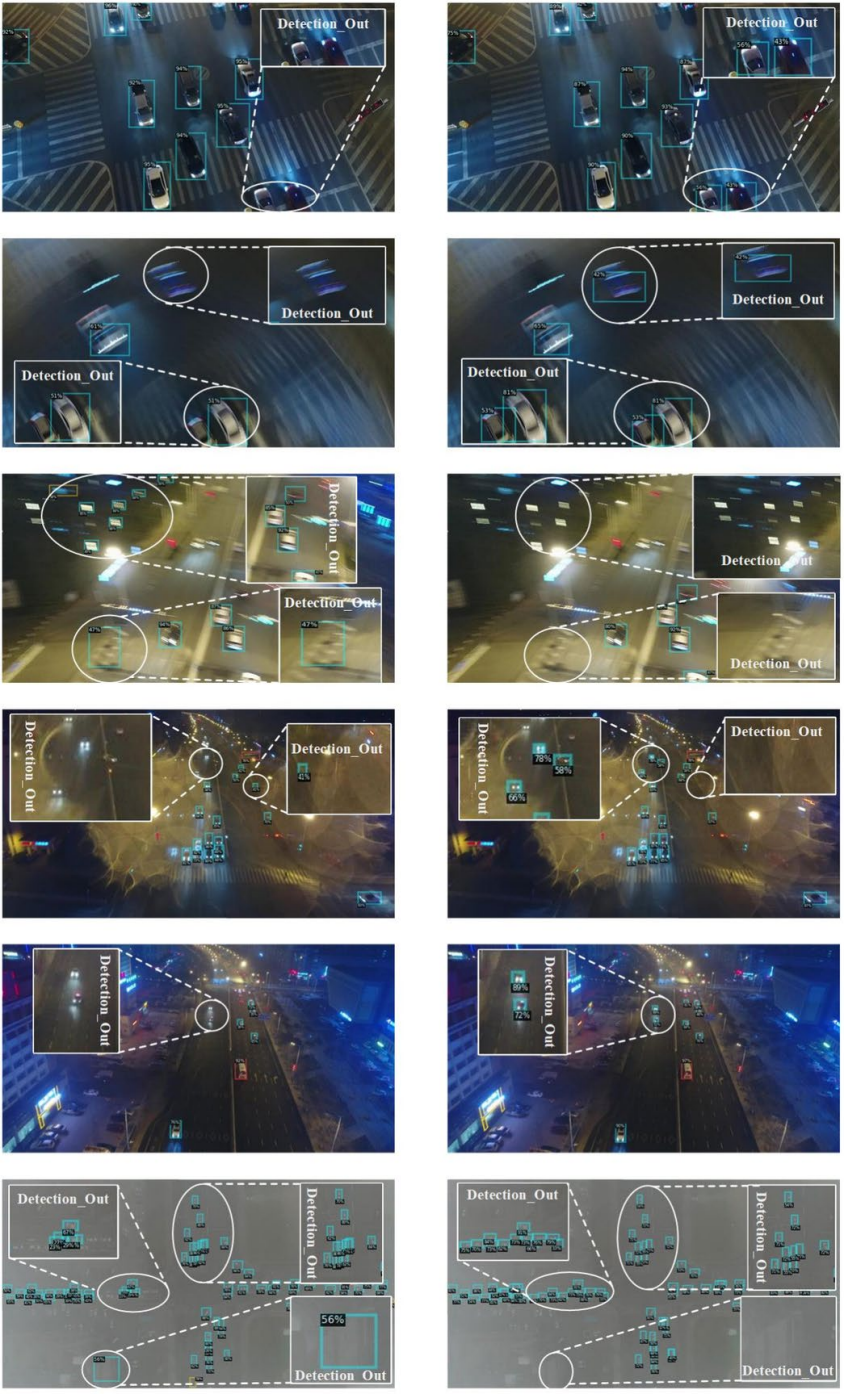
Comparison of detection results on UAVDT dataset.

Given the strong representational capabilities of both MSFA and DDCA for small objects, we integrate them cooperatively to achieve complementary multi-scale feature interaction and enhance discriminative features for small foreground targets. To further validate the effectiveness of this collaborative integration, we compare the experimental results of different module combinations: SSM with DDCA, TSM with DDCA, and MSFA with DDCA. As shown in rows 6, 7, and 8 of Table 5 on the UAVDT dataset, the results demonstrate the superior effectiveness of integrating MSFA with DDCA. As presented in row 8 and row 15 of Table 5, CMA-Net achieves an $\text {AP}$ of 67.2% on the UAVDT dataset and 62.0% on the Stanford Drone dataset, with particularly notable improvements in $\text {AP}_S$. CMA-Net enhances small object detection performance while maintaining low inference latency.

### visualization results

#### Visualization results on UAVDT dataset

To better demonstrate the advantages of our method, Figure 8 presents a visual comparison between the detection results of the Bi-FPN baseline and those of our proposed approach across several extreme scenarios from the UAVDT dataset. The left column shows the direct predictions from the Bi-FPN baseline, while the right column displays the corresponding results generated by our method. As shown in the top rows, our algorithm exhibits a stronger detection capability under challenging low-light nighttime conditions with limited resolution. The experimental results in the second and third rows reveal its superior robustness against image blur caused by camera shake. The fourth and fifth rows present outcomes under significant noise from strong artificial lighting at night, indicating our method’s enhanced effectiveness in mitigating both false positives and missed detections for small objects. Finally, the comparative results in the last row, under adverse weather conditions such as haze, demonstrate the proposed model’s practical utility and reliability in real-world application scenarios.

**Fig. 9:**
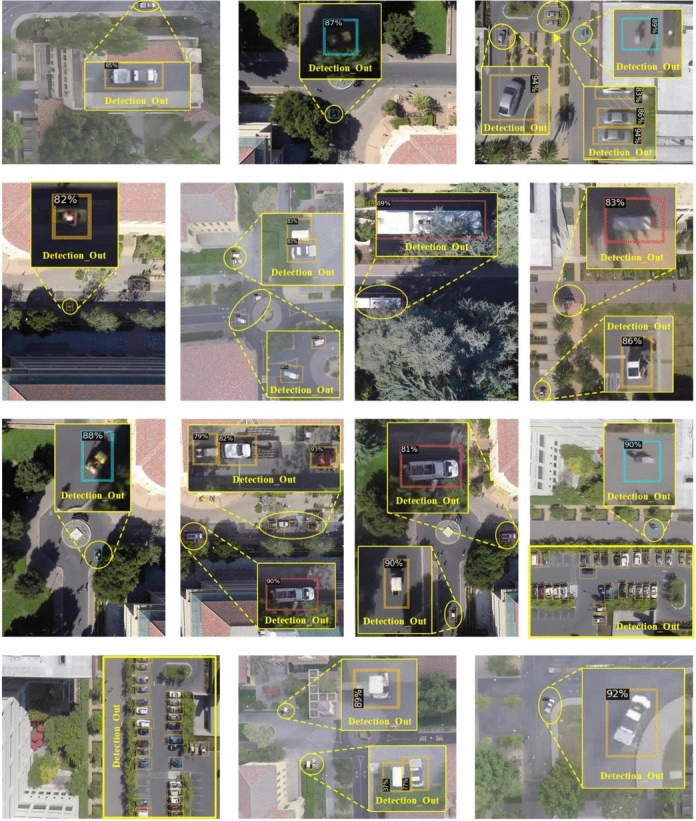
Visual detection results on Stanford Drone dataset.

#### Visualization results on stanford drone dataset

Figure 9 presents the visualization results of our proposed real-time small object detection algorithm on Stanford Drone dataset. Experimental results demonstrate that our method effectively suppresses background interference while enhancing discriminative feature representation for small objects. The algorithm accurately identifies weak and small targets in UAV imagery, particularly under challenging conditions such as foreground-background similarity and redundant information interference, exhibiting superior performance for real-time small object detection.

These visualization results provide intuitive evidence for the effectiveness of our multi-attention collaborative framework for real-time small object detection. The findings verify that our approach successfully handles information imbalance and enhances discriminative capability for small objects.

## Data Availability

The data supporting the findings of this study are publicly available. The UAVDT dataset is available at https://sites.google.com/view/grli-uavdt. The Stanford Drone dataset is available at https://cvgl.stanford.edu/projects/uav_data/.
